# Shared decision-making and client-reported dose satisfaction in a longitudinal cohort receiving injectable opioid agonist treatment (iOAT)

**DOI:** 10.1186/s13011-023-00585-4

**Published:** 2024-01-03

**Authors:** Scott Beaumont, Tianna Magel, Scott MacDonald, Scott Harrison, Martin Schechter, Eugenia Oviedo-Joekes

**Affiliations:** 1 School of Population and Public Health, University of British Columbia, 2206 East Mall, Vancouver, BC V6T 1Z3 Canada; 2 Providence Health Care, Providence Crosstown Clinic, 77 E Hastings St, Vancouver, BC V6A 2R7 Canada; 3 Urban Health and Substance Use, Providence Health Care, St. Paul’s Hospital, 1081 Burrard St, Vancouver, BC V6Z 1Y6 Canada; 4 Centre for Advancing Health Outcomes, Providence Health Care, St. Paul’s Hospital, 575-1081 Burrard St., Vancouver, BC V6Z 1Y6 Canada

**Keywords:** Shared decision making, Dose adequacy, Person-centered care, Injectable opioid agonist treatment, Opioid use disorder, Substance use

## Abstract

**Background:**

Across different types of oral Opioid Agonist Treatment for people with Opioid Use Disorder, receiving a dose that meets their needs is associated with better outcomes. Evidence also shows patients are more likely to receive an “adequate dose” when their prescribers are involving them in decision making. Neither of these findings have been studied in the context of injectable Opioid Agonist Treatment, which is the purpose of this study.

**Methods:**

This study was a retrospective analysis of an 18-month prospective longitudinal cohort study of 131 people receiving injectable Opioid Agonist Treatment. In the 18-month study, observations were collected every two months for one year, and then once more at 18 months. At 6 months, participants were asked whether their dose was satisfactory to them (outcome variable). Generalized Estimating Equations were used, to account for multiple observations from each participant. The final multivariate model was built using a stepwise approach.

**Results:**

Five hundred forty-five participant-observations were included in the analysis. Participant-observations were grouped by “dose is satisfactory” and “wants higher dose”. From unadjusted analyses, participants were less likely to report being satisfied with their dose if they: were Indigenous, had worse psychological or physical health problems, had ever attempted suicide, were younger when they first injected any drug, were a current smoker, felt troubled by drug problems, gave their medication a lower “drug liking” score, and felt that their doctor was not including them in decisions the way they wanted to be. In the final multivariate model, all previously significant associations except for “current smoker” and “troubled by drug problems” were no longer significant after the addition of the “drug liking” score.

**Conclusions:**

Patients in injectable Opioid Agonist Treatment who are not satisfied with their dose are more likely to: be troubled by drug problems, be a current smoker, and report liking their medication less than dose-satisfied patients. Prescribers’ practicing shared decision-making can help patients achieve dose-satisfaction and possibly alleviate troubles from drug problems. Additionally, receiving a satisfactory dose may be dependent on patients being able to access an opioid agonist medication (and formulation) that they *like*.

**Supplementary Information:**

The online version contains supplementary material available at 10.1186/s13011-023-00585-4.

## Background

Opioid use disorder (OUD) is a pervasive and chronic health condition which continues to be characterized by comorbid physical and mental health issues and several preventable harms, most of which—including higher risk of fatal and non-fatal overdose—are created or exacerbated by criminalization [1–3]. Evidence-based and compassionate health approaches to treat OUD, and to best support individuals’ multifaceted healthcare needs, are increasingly informed by an emerging approach: person-centered care (PCC) [4]. Through individualized and autonomy-promoting treatment, with an emphasis on enhancing the therapeutic alliance between care provider and client [5], PCC aims to shift care from the traditional paternalistic approach—in which the patient is perceived and treated as a passive recipient of health care—to one in which they are empowered to make decisions regarding their health and all aspects of their care, including treatment type, dose, and formulation [5–7]. As has been clearly established many times, including through meta-analysis [8], effective evidence-based care for OUD necessitates provision of opioid agonist treatment (OAT) through individualized and flexible dosing strategies, without “arbitrary thresholds” [9] (dose maximums). This exemplifies the relevance of PCC principles, like individualized care, in treating people with OUD, as does evidence that *patient-perceived* dose adequacy [10] is a clear predictor of treatment outcomes [11]. Further, dose adequacy for oral OAT (e.g., methadone) is best elucidated through the patients’ participation in dosage decisions [12], i.e., shared decision-making (SDM), another central aspect of PCC [4]. While emerging evidence suggests PCC is also important in state-of-the-art treatments for OUD, like injectable opioid agonist treatment (iOAT) [5, 13], patient-perceived dose adequacy, and any relationship it has with SDM, has not been studied in iOAT. Alongside the recognized ethical imperative of involving patients in their own care, evidence indicates that—across a variety of healthcare contexts—patients’ perceptions of SDM has been found to be associated with a variety of benefits, including health-related Quality of Life [14], better asthma control [15], and improved HIV-related symptoms [16]. SDM is best achieved when the client is empowered by service provider(s) to utilize the best available evidence (including the clients’ own self-expertise) to make informed decisions about their own treatment that incorporate the client’s values and needs (as defined by the client) [17, 18].

There are currently several oral opioid medications that have shown success and remain clinically relevant in helping many individuals manage their OUD. Some of these options are considered a “first line” treatment, although they often do not meet the diverse needs and preferences of an important minority of clients [19]. For those not attracted to, unable to be engaged by, or otherwise not benefiting from “first-line” treatment approaches, other alternatives exist, such as iOAT (excluding depot injections). In Canada, the iOAT medications currently available and approved for OUD include diacetylmorphine (DAM, i.e. pharmaceutical-grade heroin) and hydromorphone (HDM), both of which have demonstrated safety and efficacy in the treatment of severe OUD [20–22].

Several limitations within iOAT exist, namely, issues of accessibility and availability of medication. With few exceptions, individuals are required to be onsite at their iOAT clinic two to three times a day, requiring that they live close to the clinic—as current restrictions prevent the picking up of their medications from pharmacies. These requirements impede the delivery and accessibility of care for those who have insecure or unstable housing, live in rural or off-site locations, or would otherwise benefit from accessing medications at local pharmacies. Prescribers are also restricted in which medications (DAM and HDM) they can provide for OUD that are formulated for injection, have gone through at least one RCT, and are approved for this use by Health Canada. Several iOAT sites do not even have access to DAM, offering only HDM [23]. This lack of variety of available medications limits clients’ potential for choice in finding and accessing a medication that best fits their needs. Likewise, given the current guidelines for iOAT in Canada, total daily and per-session iOAT doses are also currently limited to maximums that have remained unchanged for many years. For example, for DAM the current maximum has remained mostly unchanged since the original Swiss trials of “heroin assisted treatment” over two decades ago, leaving little flexibility for those who may require higher doses to meet their needs [24, 25], which is especially relevant given the ever-increasing potency of criminalized opioids.

As prohibition drives and incentivizes ever-greater potency and toxicity of the criminalized drug supply [1, 2], it also leads users to incarceration, marginalization, and exposure to extralegal violence, while suppressing and restricting efforts to prevent and reverse overdoses [3]. This has fueled the ongoing drug poisoning crisis which continues to claim the lives of many in North America, including first-time, infrequent, and non-opioid users [26]. Under these conditions, treatment for OUD is currently one of the only interventions available (if not accessible) for an important subset of people at risk of overdose (i.e., people with OUD). There is, therefore, a clear need for better understanding of factors outside of medication alone that impact how people with OUD access care and treatment.

For patients with OUD, individualized treatment options and SDM around medication and dose has shown evidence of increased satisfaction with care and improvements in overall quality of life scoring [27, 28]. Oral OAT clients’ subjective experiences of the adequacy of their dose has also been studied in the context of treatment outcomes in which adequate doses have been linked to increased retention, lower risk of infectious disease, and reduced criminalized and poly-substance use [29–31]. In the context of methadone, there is robust evidence supporting the practice of tailoring the medication to the client’s needs and offering flexible dosing options [32]. Trujols et al., found that clients’ perception of their involvement in the dose decision making process was the only predictor that demonstrated an association with clients’ dose adequacy status [12]. These findings align with perspectives of iOAT providers and stakeholders who emphasize the importance of client autonomy and acknowledge the difficulties of providing PCC within the constraints of current system-level barriers [33].

Despite this evidence, dose adequacy has not been studied outside of the context of “first line” treatments (i.e. oral OAT such as methadone and buprenorphine) and so there is little empirical academic work examining dose adequacy perceptions among those receiving iOAT. With the continued expansion of iOAT in Canada and across Europe, there is a fundamental need to study how to optimize iOAT programs such that they become increasingly equitable and accessible. Client dose adequacy perceptions can help optimize treatment outcomes through the integration of personalized treatment and SDM. The present study aims to explore the differences between clients reporting dose satisfaction vs. dissatisfaction, as well as clients’ perceptions of their involvement in treatment decision-making and its respective association with self-reported dose satisfaction.

## Methods

The present study is a secondary retrospective analysis which examined data from clients receiving iOAT at a clinic in the Downtown Eastside neighborhood of Vancouver, Canada. All participants were enrolled in the longitudinal Research on the Utilization of Therapeutic Hydromorphone (RUTH) study and the Study to Assess Longer-term Opioid Medication Effectiveness (SALOME). Detailed methods on both studies have previously been reported elsewhere [34–36]. Briefly, SALOME was a randomized non-inferiority trial assessing the efficacy of injectable hydromorphone and diacetylmorphine. Of those enrolled in SALOME, 131 participants transitioned to the open-label 18-month longitudinal inferential RUTH study from 2016–2018. Data for RUTH was collected every two months for one year and then once at 18 months for a total of eight study timepoints. Baseline demographic and lifetime history variables were collected prior to SALOME randomization and were entirely based on participants’ self-report. All other variables were collected at each RUTH study timepoint except for client dose satisfaction, which was not included until the 6-month time point. *Participant reported dose satisfaction* was ascertained solely from participants’ responses to the question: “Were you satisfied with the average daily dose prescribed to you in the last 30 days?”. Participant response options included “Yes”, “No, I wanted a higher dose”, or “No, I wanted a lower dose”. Given the limited responses indicating wanting a “lower dose” (*N* = 5 observations across all time points), these observations were considered as not applicable (N/A).

At each RUTH time point, several standardized questionnaires were completed by participants to garner information on self-reported physical and mental wellbeing, nicotine dependence, criminalized use of substances outside of treatment, satisfaction with medication dose, and client perceptions of health care providers’ communication and SDM. Questionnaires included the: Maudsley Addiction Profile (MAP) [37], European Addiction Severity Index (EuropASI) [38], Communication Assessment Tool (CAT) [39], Client Satisfaction Questionnaire (CSQ) [40], Opiate Treatment Index (OTI) [41], and Fagerström Test for Nicotine Dependence (FTND) [42]. From the OTI, a universal total health score was created by removing the Gynecological sub-score (to avoid inflating the score for the subset of participants for whom those questions are relevant).

### Data analysis

Unadjusted and adjusted analysis utilized Generalized Estimating Equations (GEE). As a natural extension of Generalized Linear Models, GEE is suitable for estimating population effects using correlated longitudinal, within-subject, repeated measures sample data. Complete cases were utilized for analysis leaving a total of 545 participant-observations for the timeframe of 6 to 18 months. Model building used a stepwise comparison approach whereby variable “blocks” or “domains” were added systematically to the model. Final block inclusion and model selection was determined by a combination of theoretically informed key variables (e.g. gender, age) and theoretically or empirically relevant variables with an independent association with dose satisfaction (in an unadjusted analysis) whose inclusion in the multivariate model resulted in a reduction to the Quasi Information Criterion (QIC), a modified version of Akaike’s Information Criterion appropriate for model selection in GEE [43]. Multiple imputation, using multivariate imputation by chained equations (MICE), was conducted to address missing data. Non-outcome variables were considered eligible for imputation if less than 50% of observations were missing for the respective questionnaire. Final scoring collated generated imputed scores by taking median scores across multiple imputations as the final imputed value for missing participant observations. All statistics and visual summaries were completed using the statistical software R (Version 4.3.0) via the RStudio integrated development environment (2023.06.0 + 421 “Mountain Hydrangea” Release).

## Results

Of the 131 participants in RUTH, a total of 129 participants (2 deceased by 6 months) were included in this analysis, and after removal of observations with missing outcome data or with responses of “wants lower dose”, a total of 545 total participant observations remained. All 129 possible participants have at least one observation included in the analysis. Participant characteristics separated by dose adequacy are presented in Table 1. Based on self-report, participants were on average 45.30 years old (SD = 8.91), 28% were women (including cisgender and transgender women), 29% were Indigenous (Métis, Inuit, First Nations), and mean reported number of days injecting "heroin" in the previous month (while in treatment) was 4.35 days (SD = 8.25).

**Table 1:** Participant characteristics grouped by patient-reported dose satisfaction (*N* = participant-observations), with significant *p*-values from unadjusted generalized estimating equations

Characteristic	Patient-reported Dose Satisfaction Observations		
	Overall *N* = 545^a,d^	Satisfied with Dose *N* = 423^a,d^	Wants Higher Dose *N* = 122^a,d^
**Age**^**b**^	45.30 (8.91)	45.86 (8.69)	43.37 (9.43)
**Gender**^**b**^			
**Female**	155 (28%)	110 (26%)	45 (37%)
**Male**	390 (72%)	313 (74%)	77 (63%)
**First Nations, Métis, or Inuit**^**b**^			
**No**	385 (71%)	311 (74%)	74 (61%)
Yes*	**160 (29%)**	**112 (26%)**	**48 (39%)**
**Education**^**b**^			
**Less than high school**	230 (42%)	172 (41%)	58 (48%)
**High School**	138 (25%)	103 (24%)	35 (29%)
**At least some post-secondary**	177 (32%)	148 (35%)	29 (24%)
**EQ5D-CAN Score**^**c**^	0.79, (0.21)	0.80, (0.21)	0.76, (0.21)
OTI Total Health Score (without gynecological subscale)** ^**c**^	**46.88 (25.98)**	**44.51 (26.36)**	**54.96 (22.97)**
**Ever Attempted Suicide**^**b**^			
**No**	398 (74%)	327 (78%)	71 (59%)
Yes*	**142 (26%)**	**92 (22%)**	**50 (41%)**
Age First Injected Any Drug* ^**b**^	**23.17 (8.06)**	**23.68 (8.18)**	**21.40 (7.38)**
Days with heroin use in past month* ^**c**^	**4.35 (8.25)**	**3.76 (7.62)**	**6.38 (9.90)**
**Number of days (out of past 30) patient smoked crack cocaine** ^**c**^	5.14 (10.28)	5.00 (10.29)	5.63 (10.29)
**FTND **^**c**^			
**Non-Smoker**	66 (12%)	63 (15%)	3 (2.5%)
Smoker**	**478 (88%)**	**360 (85%)**	**118 (98%)**
**Whether patients were troubled to any extent by any drug problems**^**3**^			
**No**	257 (47%)	218 (52%)	39 (32%)
Yes**	**288 (53%)**	**205 (48%)**	**83 (68%)**
MAP Psychological Health Score** ^**c**^	**8.49 (7.37)**	**7.68 (6.95)**	**11.29 (8.12)**
**Average Daily Dose Prescribed**^**c**^	562.59 (261.58)	555.93 (257.78)	585.91 (274.28)
**Patient's rating of doctor involving them in decisions as much as they wanted**^**c**^			
**Less Than Excellent**	275 (52%)	199 (48%)	76 (65%)
Excellent**	**254 (48%)**	**213 (52%)**	**41 (35%)**
Drug Liking VAS*** ^**c**^	**74.22 (24.17)**	**78.08 (21.08)**	**61.02 (29.05)**

Values are bolded if there is a significant (*p* < 0.05) difference in means between dose-satisfaction groups (for continuous variables) or significant difference in distribution of dose-satisfaction status between one or more levels of a categorical variable and the (not bolded) reference level

*Abbreviations*: *EQ5D-CAN* Canadian version of the EuroQol 5 Dimension descriptive system for health-related quality of life, *OTI* Opiate Treatment Index, *FTND* Fagerström Test of Nicotine Dependence, *MAP* Maudsley Addiction Profile, *VAS* Visual Assessment Scale

^*^p < 0.05

^**^p < 0.01

^***^p < 0.001

^a ^n (%); mean (SD)

^b ^Variable collected at SALOME baseline

^c ^Variable reflects prior 30 days at the time of RUTH study visit

^d ^The N here is observations and each participant has up to 5 observations represented, therefore, some participants have observations) represented in both -groups

### Dose adequacy groups—unadjusted analysis

Among the 545 observations where participants indicated their dose satisfaction, 77.6% (423) indicated that participants at the associated time points were satisfied with their dose, versus 22.4% (122) observations of participants who reported dissatisfaction with their dose and wanting a higher dose at those time points. In unadjusted analyses (see Table 1*,* and see Appendix 1 for additional variables and additional statistics such as medians and inter-quartile ranges), Indigeneity, OTI total health score, age of first use by injection of any criminalized drug, “heroin” use in the previous 30 days (while in treatment), and current smoking status were statistically significant (*p* < 0.05). Higher Maudsley Addiction Profile (MAP) Psychological Health scores (indicating worse mental health), lower scores from participants when rating how much their doctor involves them in treatment decisions (SDM), feeling more troubled by problems associated with criminalized drug use, and lower “[iOAT] drug liking” scores were also found to be associated with participant reported iOAT dose dissatisfaction.

### Adjusted multivariate dose adequacy analysis

In adjusted analyses (see Table 2 for final adjusted model and see Appendix 2 for full step-wise model-building process) no sociodemographic variables (Step 1) were associated with participant reported iOAT dose adequacy. In Step 2, a variable related to physical health was added into the adjusted model: the OTI Total Health score, which was associated with dose-satisfaction status, with an adjusted odds ratio [AOR] of 1.01 (95% CI: 1.00, 1.02) greater likelihood of “wants a higher dose” status with each increase in OTI Total Health score (indicating worse overall health). This association did not persist through the next steps. No variables related to Mental Health showed an association with dose-satisfaction status, across any steps of the model, when adjusting for other variables. The Quality of Life measure, EQ5D-CAN score, did not show any association with dose-satisfaction in the adjusted models. Participant’s current smoker vs non-smoker status (AOR: 3.69; 95% CI: 1.23, 11.1), and participant self-reported “feeling troubled with problems associated with criminalized substance use” as opposed to untroubled (AOR: 2.05; 95% CI: 1.06, 3.95), remained statistically significant from their introduction in Step 5 until the final model in Step 7. When introduced in Step 6, clients who rated their doctor as “excellent” at SDM (involving the client in decisions as much as the client wants), were less likely to also report dissatisfaction due to wanting a higher dose (AOR: 0.652; 95% CI 0.453, 0.938). This variable did not stay significant after the last step, with the introduction of the final variable: patient-reported “Liking the (prescribed) drug” from the Visual Assessment Scale, where each increase in rating (which ranged from 0–100) was associated with a lower likelihood of the client wanting a higher dose (AOR 0.983; 95% CI 0.973, 0.993). Compared to a person who rates their iOAT drug as 0 for “drug liking”, a person who rates it as 100 is only 81.71% as likely to report that they are unsatisfied with their dose due to wanting a higher dose. Variable inclusion at each step was informed by reductions in the Quasi Information Criterion score (see Appendix 2), suggesting the final model had the best fit to the data: balanced between explaining the greatest amount of variability while minimizing loss of parsimony from adding more variables [44].

**Table 2:** Final multivariate generalized estimating equation model (from stepwise model building and comparison process), including adjusted odds ratios of iOAT clients’ likelihood of reporting dissatisfaction with their dose, shown next to unadjusted odds ratios

**Block/Step**	**Variable (and Variable Level)**		**Bivariate OR (95% CI)**	**Multivariate AOR (95% CI)**
*1. Socio-demographics*	Age^a^		0.97 (0.94, 1.01)	0.996 (0.96, 1.03)
	Gender (self-identified)	Woman^b^	—	*—*
		Man	0.61 (0.32, 1.16)	0.818 (0.40, 1.66)
	First Nations, Métis, or Inuit	No^b^	—	—
		Yes	**1.89 (1.00, 3.56)**	1.38 (0.67, 2.85)
	Education	Some High School or less^b^	—	—
		High School Diploma	1.10 (0.53, 2.27)	1.51 (0.71, 3.22)
		≥ Some post-secondary	0.62 (0.31, 1.27)	0.90 (0.43, 1.87)
*2. Physical Health*	Opiate Treatment Index—Health Section Total Score^c^		**1.01 (1.00, 1.02)**	1.01 (0.99, 1.02)
*3. Mental Health*	Ever attempted suicide	No^b^	—	—
		Yes	**2.29 (1.20, 4.35)**	1.39 (0.69, 2.81)
	MAP Psychological Score		**1.04 (1.01, 1.08)**	1.01 (0.98, 1.04)
*4. Quality of Life*	EQ5D-CAN Score		0.41 (0.11, 1.58)	1.00 (0.22, 4.44)
*5. Substance Use and Treatment*	Average Daily Prescribed iOAT dose^d^		1.00 (1.00, 1.00)	1.00 (1.00, 1.00)
	Days with heroin use in past month		**1.02 (1.00, 1.05)**	1.00 (0.97, 1.04)
	Days with crack cocaine use in past month		0.99 (0.96, 1.02)	1.01 (0.99, 1.04)
	FTND: Current smoker	Non-smoker^b^	—	—
		Smoker	**4.48 (1.66, 12.1)**	**1.17 (1.06, 1.30)**
	How troubled client reports feeling about problems associated with criminalized drug use	Untroubled^b^	—	—
		Troubled	**2.21 (1.21, 4.01)**	**2.06 (1.12, 3.79)**
*6. Shared Decision Making*	Client rating of doctor involving them in decisions as much as the client wants	Less than excellent^b^	—	—
		Excellent	**0.65 (0.47, 0.88)**	0.81 (0.61, 1.08)
*7. Drug Liking*	Visual Assessment Scale of "Liking the (prescribed) Drug"		**0.98 (0.97, 0.99)**	**0.98 (0.97, 0.99)**

Values are bolded if there is a significant (*p* < 0.05) difference in means between dose-satisfaction groups (for continuous variables) or significant difference in distribution of dose-satisfaction status between one or more levels of a categorical variable and the (not bolded) reference level

*Abbreviations*: *EQ5D-CAN* Canadian version of the EuroQol 5 Dimension descriptive system for health-related quality of life, *OTI* Opiate Treatment Index, *FTND* Fagerström Test of Nicotine Dependence, *MAP* Maudsley Addiction Profile, *VAS* Visual Assessment Scale

^a^ per 1 year increase

^b^ reference group for both OR and AOR

^c^ (minus Gynecological subscale)

^d^ per 1 mg increase in diacetylmorphine-equivalent-mg

## Discussion

The quantitative evidence presented within this study demonstrates that dose satisfaction is an important consideration in the provision of iOAT, as has been found with dose adequacy in other forms of OAT [11, 12, 45]. This is clear from many of the findings of this study, but perhaps most obviously it is shown via participants who, when they are not satisfied with their dose, are also more likely to report being troubled by problems related to criminalized drug use. One of the main goals of all treatment for OUD is to reduce clients’ problems from/with criminalized drug use, which can stem from frequency of use, but also from issues associated with obtaining criminalized drugs (and the money required to buy them), and how/when/where/with whom criminalized drugs can be used. This inherent complexity is reflected in the finding that clients’ perception of troubles from criminalized drug use has a stronger association with dose satisfaction than actual frequency of criminalized opioid (“heroin”) use. Further, feeling "troubled by problems" can likely be considered a proxy for stress levels, and higher levels of stress are a known predictor of dropout from OUD treatment [46], meaning this is a particularly vulnerable group of clients.

Whether clients are smokers or not also emerged as a strong predictor of dose-satisfaction status. Explanations include (but are not limited to): greater tolerance to the euphoric effects of opioids to increased stress levels from inadequate dosing and increased withdrawal symptoms. Since stress is a principal factor promoting tobacco use [47], inadequate dosing would make it less likely that those participants would be empowered to choose—and succeed in—managing, reducing, or quitting smoking. Adequate dosing is an inherent part of effective treatment [48], and participants who smoke tobacco and/or who experienced more troubles from problems associated with criminalized drugs were more likely to also be receiving an inadequate dose, and therefore inadequate treatment.

Our results also indicate that iOAT clients’ perception of their doctors involving them in decisions (i.e. sharing decision-making power) was significantly associated with clients’ dose satisfaction status in both unadjusted modelling and when first introduced to the stepwise adjusted model. Clients who like the drug they’re receiving are more likely to feel satisfied with the dose, whereas clients who do not like it as much are less likely to feel satisfied with the dose, and—once this is accounted for—whether shared decision-making is happening or not becomes less important. This suggests that restrictions on which drugs are available to clients likely impacts treatment attractiveness and engagement, something which has already been explicitly stated by people with OUD [49]. Since prescribers (and programs) are currently restricted in terms of which injectable opioid agonists they can provide for iOAT, there is a corresponding limit to how many clients will be able to access the opioid agonist they would like. Additionally, providers who have made efforts to promote client autonomy have highlighted feeling constrained by system-level restrictions and regulations (e.g. policies, governing structures, etc., that establish, facilitate, and enforce how iOAT is delivered in Canada) [33] that obstruct efforts and the overall promotion of PCC. Existing research has looked at dose adequacy for patients on methadone maintenance treatment, and found that “patients’ perception of participation in methadone dosage decisions” was the only variable independently associated with patients perceiving their methadone dose to be inadequate [12]. Promotion of individual autonomy and PCC can be further constrained by medical professionals’ reactions to patients demonstrating in-depth knowledge about their own health conditions, medications, and treatments. Patients who attempt to self-advocate using relevant expertise encounter problems in interactions with healthcare providers, who often react to patients’ biomedically and/or experientially informed self-advocacy by reasserting control over medication and medical decisions in ways which run counter to patients’ preferences and needs, resulting in worse outcomes [50]. Until iOAT clients are regularly included in decisions around and able to access drugs that they do “like” the effects of, achieving true dose satisfaction for a subset of iOAT clients will be as out-of-reach as those preferred drugs are.

The lack of association between dose-satisfaction status and the actual dose prescribed is congruent with other literature from PCC-informed programs [12]. Research examining clients’ typical iOAT doses found that, within the spectrum of available doses, the majority of clients receive doses in the middle range with a small number (~ 5%) of individuals requiring higher or lower doses (Unpublished observations Magel et al.). These findings emphasize the importance of offering individualized flexible [8] dosing for opioid agonists. Individualized care and SDM are two fundamental aspects of PCC, which has been shown to promote client autonomy [51] increase engagement with treatment [52], and reduce criminalized drug use, addiction severity, and psychiatric problems [53]. These are also integral parts in the building of a therapeutic relationship between provider and client, establishing a foundation in which clients are able to feel comfortable and safe opening up about their needs. This further encourages clients to be involved in the decisions around care, maximizing the potential for the provision of care that is responsive to clients’ individual needs, preferences, histories, and disabilities [5, 33].

Current medication and dose concerns are, understandably, centered around client safety. In Canada, iOAT clients undergo a rigorous three day induction protocol, and doses are usually restricted to a maximum of 1000 mg and 500 mg per day (and 400 mg and 200 mg per session) for diacetylmorphine and hydromorphone, respectively [24, 35]. iOAT clinical trial data, comparing hydromorphone and diacetylmorphine, recorded a total of 29 (5 in hydromorphone and 24 in diacetylmorphine) serious adverse events (SAEs) among 41,027 and 44,424 recorded hydromorphone and diacetylmorphine injections, respectively [35]. All SAE’s were safely mitigated [21]. A case study from Tas et al. also found that diacetylmorphine injections caused respiratory regulation abnormalities for a man on long-term injectable diacetylmorphine maintenance treatment, but that even for just one subject, there was an inconsistent relationship between opioid dose and overdose risk (respiratory depression) [54]. This suggests that inflexible restrictions on changes to—especially increases beyond the typical “maximum”—doses may not always provide benefits to all client’s safety, given the wide variation in tolerances and responses to opioid agonists both within and between patients. Indeed, there is documented evidence of methadone clients whose effective doses have been found to go up to over 700 mg [9]. Most importantly, the risk of overdose for a client should be weighed against the risk of that client turning to criminalized opioids to supplement their OAT, and then potentially overdosing from an unknown purity substance in a non-clinical setting. This should also be considered when discussing the possibility of medication diversion, a concern often cited to justify current barriers (e.g. restrictions on take home doses) to the provision of greater flexibility and accessibility of OAT. Evidence suggests, however, that diversion has many positive effects and plays an important role in keeping people alive during the current unintentional drug poisoning crisis driven by the toxic criminalized drug supply [55]. There is little to no rigorous evidence suggesting that diverted OAT medications are used by anyone other than already-regular users of criminalized opioids, or used for reasons other than those individuals trying to manage their own OUD in the absence of care that is attractive, accessible, and effective for them. It is clear that implementation of PCC for OUD would necessitate not just greater shared decision-making by prescribers, but also more flexibility around: restrictions on which medication type (e.g. offering fentanyl and/or analogues to those who prefer them), formulation (e.g. options for smoking or snorting appropriately formulated medications), doses (e.g. increased range of offered doses without rigid maximums), and accessibility (e.g. offering take-home doses, including via delivery and via local pharmacies).

### Limitations

The unique nature of this cohort poses several potential limitations, including the generalizability of findings given the uniqueness of iOAT clients and the respective use of baseline data from the SALOME clinical trial. However, given the criteria used to enroll both SALOME and RUTH participants, it is anticipated that findings from this study are reflective of individuals with opioid use disorder and thus, generalizable to this population. It is worth noting that at one point during the study, a DAM shortage took place, resulting in participants temporarily being switched to HDM. It is possible that, among those whose medications had to be switched, perceived dose adequacy may have acted as a proxy for “drug liking” during this time. However, this shortage is unlikely to have resulted in significant changes to findings as its duration was less than 2 months and all individuals were returned to their preferred medication. The inability to examine observations where individuals indicated they wanted a lower dose, and therefore their exclusion from the analysis, was a limitation in comparing groups. Still, given the dearth of individuals in this group (i.e.. *n* = 5), removal of these observations likely did not impact our final findings. Further, individuals receiving iOAT have autonomy to choose whether they wish to use the entire amount of their medication and are not required to use the full dose if they do not wish to. Perceptions of SDM were also restricted to those of clients. Future research could explore integrating both client and iOAT provider perspectives in the analysis of SDM.

An additional limitation of our analysis is that there are many additional intersecting psychological and sociological factors that may need further exploration. The way a participant perceives their engagement in shared-decision making or their dose adequacy could be shaped by their unique psychology, including their externalizing tendencies (e.g. opposition to authority; feelings of resentment or anger), as well as the sociological context shaping their psychological and behavioral tendencies. Following this example, what is often perceived or labelled as "externalizing behaviors" cannot be considered in isolation from experiences of abuse, oppression, and marginalization at the hands of authority figures and institutions. Therefore, future research could further analyze factors including: Indigeneity, gendered and racialized oppression, poor mental health, disability—to name a few—and their complex and varied intersections [56]. For example, given centuries of ongoing settler-colonial occupation, genocide [57], and other forms of oppression, it is well documented that Indigenous people have unique and worse experiences with navigating colonial healthcare systems which impacts their health outcomes, behaviors, perceptions, and expectations [58].

A different type of limitation is that this study’s analysis plan was not preregistered, a process which would have demonstrated proof of confirmatory hypothesis testing, allowed for improved calibration of uncertainty parameters, and insured the foundational assumptions of the various statistical tests were not undermined [59]. Although there were a priori hypotheses informed by prior research, without ironclad empirical proof of such, it would be justified for readers to treat these results as the results of an exploratory or post-hoc analysis.

The study data used for this secondary analysis was not originally designed to ensure adequate power for this analysis. Looking at the relationship between methadone patients’ participation in dose decisions and their dose adequacy status, Trujols et al. [12] provides an odds ratio, which can be converted to an effect size [60]. Given the similarities of Trujols et al.’s work to our study, it is reasonable to treat this effect size much like a Bayesian prior for what we might expect. Indeed, using this we would anticipate a power of 99%. That we did not detect a difference in our final model confounds this expectation. There are a number of possible explanations for the discrepancy, including: important differences between Trujols et al.’s validated ODAS scores for determining dose-adequacy versus our use of a post-hoc analysis comprised of a single item with two response options, and differences between Trujols et al.’s question about patients’ perception of involvement specifically in *dose* decision-making versus our question which asked about *overall* shared decision-making.

The differences between the outcome measures is particularly salient, as single item scales (like the one used here to measure patient-perceived dose-satisfaction): tend to have lower content validity (inability to adequately capture the construct), have fewer points of discrimination (and therefore less sensitivity), and lack a way to measure internal-consistency or reliability (computing Cronbach’s alpha, the most common measure of reliability, requires at least two items).

Given these limitations, we are currently exploring new research with the aim of addressing and answering emerging questions. We intend to develop an adapted scale for measuring dose adequacy specific to iOAT clients, which will involve relying on the foundational work of Gonzalez-Saiz et al. in developing the theoretical construct of dose adequacy and creating the Opiate Dosage Adequacy Scale [10]. Furthermore, informed by our earlier studies, we intend to pilot a question specifically about patients’ perception of participation in *dose* decision-making, in addition to overall shared decision-making.

## Conclusion

To our knowledge, this is the first study to analyze iOAT clients’ self-reported dose-satisfaction while also examining other factors associated with participants’ dose-satisfaction status. Findings about the importance of client perceived dose satisfaction, how much the client likes the drug, and shared decision-making supports previous research on similar populations that has emphasized the benefits of PCC and the importance of promoting clients’ autonomy. Study findings on clients’ perceptions of dose satisfaction and drug liking highlight important aspects of client care that can be considered in the pragmatic implementation and evaluation of PCC and SDM in OUD treatment settings.

### Supplementary Information

**Additional file 1.** Table of participant characteristics and table of full stepwise block model.

## Data Availability

The datasets generated and/or analysed during the current study are not publicly available due to potential participant identification but are available from the corresponding author on reasonable request.

## Abbreviations

CSQ: Client satisfaction questionnaire
EuropASI: European addiction severity index
FTND: Fagerström test for nicotine dependence
GEE: Generalized estimating equation
iOAT: Injectable opioid agonist treatment
OAT: Opioid agonist treatment
OTI: Opiate treatment index
OUD: Opioid use disorder
MAP: Maudsley addiction profile
MICE: Multivariate imputation by chained equations
PCC: Person-centered care
RUTH: Research on the Utilization of Therapeutic Hydromorphone
SAE: Severe adverse events
SDM: Shared decision-making
SALOME: Study to Assess Longer-term Opioid Medication Effectiveness
DAM: Diacetylmorphine
HDM: Hydromorphone
