# Supplementary material for: Shared decision-making and client-reported dose satisfaction in a longitudinal cohort receiving injectable opioid agonist treatment (iOAT)
Source: Subst Abuse Treat Prev Policy. 2024 Jan 3;19:1. doi: 10.1186/s13011-023-00585-4 (PMC10763140; doi:10.1186/s13011-023-00585-4)
Supplement: Supplementary file 1 — Additional file 1. Table of participant characteristics and table of full stepwise block model. [file 13011_2023_585_MOESM1_ESM.docx]

**APPENDIX**

**Appendix Table 1.** Characteristics Grouped by Patient-Reported Dose Satisfaction status, with Odds Ratios from associated bivariate GEEGLM model

| **Characteristic** | Patient-Reported Dose Satisfaction | | | **OR^2^** | **95% CI^2^** | **p-value** |
| --- | --- | --- | --- | --- | --- | --- |
|  | **Overall**, N = 545^1^ | **Dose is Adequate**, N = 423^1^ | **Wants Higher Dose**, N = 122^1^ |  |  |  |
| **Age** |  |  |  | 0.97 | 0.94, 1.01 | 0.11 |
| Mean, (SD) | 45.30, (8.91) | 45.86, (8.69) | 43.37, (9.43) |  |  |  |
| Median, [IQR] | 46.00, [41.00, 51.00] | 46.00, [42.00, 52.00] | 44.00, [38.00, 50.00] |  |  |  |
| **Age Group** |  |  |  |  |  |  |
| <35 | 39 (7.2%) | 25 (5.9%) | 14 (11%) | — | — |  |
| 35-50 | 227 (42%) | 168 (40%) | 59 (48%) | 0.56 | 0.18, 1.69 | 0.3 |
| >50 | 279 (51%) | 230 (54%) | 49 (40%) | 0.38 | 0.12, 1.16 | 0.088 |
| **Gender** |  |  |  |  |  |  |
| Female | 155 (28%) | 110 (26%) | 45 (37%) | — | — |  |
| Male | 390 (72%) | 313 (74%) | 77 (63%) | 0.61 | 0.32, 1.16 | 0.13 |
| **First Nations, Métis, or Inuit** |  |  |  |  |  |  |
| No | 385 (71%) | 311 (74%) | 74 (61%) | — | — |  |
| Yes | 160 (29%) | 112 (26%) | 48 (39%) | 1.89 | 1.00, 3.56 | **0.050** |
| **Education** |  |  |  |  |  |  |
| Less than high school | 230 (42%) | 172 (41%) | 58 (48%) | — | — |  |
| High School | 138 (25%) | 103 (24%) | 35 (29%) | 1.10 | 0.53, 2.27 | 0.8 |
| At least some post-secondary | 177 (32%) | 148 (35%) | 29 (24%) | 0.62 | 0.31, 1.27 | 0.2 |
| **Chronic Medical Problems which Interfere with Life** |  |  |  |  |  |  |
| No | 248 (46%) | 200 (47%) | 48 (39%) | — | — |  |
| Yes | 297 (54%) | 223 (53%) | 74 (61%) | 1.38 | 0.74, 2.57 | 0.3 |
| **EQ5D-CAN Score** |  |  |  | 0.41 | 0.11, 1.58 | 0.2 |
| Mean, (SD) | 0.79, (0.21) | 0.80, (0.21) | 0.76, (0.21) |  |  |  |
| Median, [IQR] | 0.83, [0.66, 1.00] | 0.84, [0.71, 1.00] | 0.78, [0.60, 1.00] |  |  |  |
| Missing | 5 | 1 | 4 |  |  |  |
| **OTI Total Health Score (without gynecological subscale)** |  |  |  | 1.01 | 1.00, 1.02 | **0.004** |
| Mean, (SD) | 46.88, (25.98) | 44.51, (26.36) | 54.96, (22.97) |  |  |  |
| Median, [IQR] | 45.00, [29.00, 63.00] | 41.00, [26.75, 61.00] | 55.00, [41.00, 68.75] |  |  |  |
| Missing | 7 | 7 | 0 |  |  |  |
| **Ever Attempted Suicide** |  |  |  |  |  |  |
| No | 398 (74%) | 327 (78%) | 71 (59%) | — | — |  |
| Yes | 142 (26%) | 92 (22%) | 50 (41%) | 2.29 | 1.20, 4.35 | **0.012** |
| Missing | 5 | 4 | 1 |  |  |  |
| **Age First Injected Any Drug** |  |  |  | 0.96 | 0.92, 1.00 | **0.046** |
| Mean, (SD) | 23.17, (8.06) | 23.68, (8.18) | 21.40, (7.38) |  |  |  |
| Median, [IQR] | 22.00, [17.00, 28.00] | 23.00, [17.00, 29.00] | 19.00, [16.00, 25.75] |  |  |  |
| **Number of days (out of past 30) patient injected heroin (baseline)** |  |  |  | 0.98 | 0.94, 1.02 | 0.4 |
| Mean, (SD) | 25.92, (7.48) | 26.21, (7.30) | 24.95, (8.02) |  |  |  |
| Median, [IQR] | 30.00, [25.00, 30.00] | 30.00, [25.00, 30.00] | 30.00, [22.50, 30.00] |  |  |  |
| Missing | 29 | 24 | 5 |  |  |  |
| **Days with heroin use in past month (in treatment)** |  |  |  | 1.02 | 1.00, 1.05 | **0.035** |
| Mean, (SD) | 4.35, (8.25) | 3.76, (7.62) | 6.38, (9.90) |  |  |  |
| Median, [IQR] | 0.00, [0.00, 4.00] | 0.00, [0.00, 3.00] | 1.00, [0.00, 7.75] |  |  |  |
| Missing | 1 | 1 | 0 |  |  |  |
| **Number of days (out of past 30) patient injected cocaine (baseline)** |  |  |  | 1.05 | 1.00, 1.09 | **0.029** |
| Mean, (SD) | 12.21, (10.54) | 10.57, (9.21) | 15.66, (12.28) |  |  |  |
| Median, [IQR] | 10.00, [2.00, 23.75] | 10.00, [2.00, 15.00] | 10.00, [3.00, 30.00] |  |  |  |
| Missing | 319 | 270 | 49 |  |  |  |
| **Days with cocaine powder use in past month (in treatment)** |  |  |  | 1.02 | 0.99, 1.06 | 0.2 |
| Mean, (SD) | 1.77, (6.10) | 1.43, (5.34) | 2.94, (8.12) |  |  |  |
| Median, [IQR] | 0.00, [0.00, 0.00] | 0.00, [0.00, 0.00] | 0.00, [0.00, 0.00] |  |  |  |
| Missing | 1 | 1 | 0 |  |  |  |
| **Number of days (out of past 30) patient smoked crack cocaine (baseline)** |  |  |  | 1.03 | 1.00, 1.05 | **0.033** |
| Mean, (SD) | 9.58, (12.44) | 8.68, (12.02) | 12.72, (13.38) |  |  |  |
| Median, [IQR] | 1.00, [0.00, 20.00] | 1.00, [0.00, 15.00] | 5.00, [1.00, 30.00] |  |  |  |
| **Days with crack cocaine use in past month (in treatment)** |  |  |  | 0.99 | 0.96, 1.02 | 0.6 |
| Mean, (SD) | 5.14, (10.28) | 5.00, (10.29) | 5.63, (10.29) |  |  |  |
| Median, [IQR] | 0.00, [0.00, 2.00] | 0.00, [0.00, 1.00] | 0.00, [0.00, 4.00] |  |  |  |
| Missing | 4 | 3 | 1 |  |  |  |
| **Number of days (out of past 30) patient injected amphetamines (baseline)** |  |  |  | 0.99 | 0.94, 1.04 | 0.8 |
| Mean, (SD) | 7.07, (9.30) | 7.15, (9.46) | 6.78, (8.82) |  |  |  |
| Median, [IQR] | 2.85, [1.00, 10.00] | 2.50, [1.00, 10.00] | 2.85, [2.00, 7.00] |  |  |  |
| Missing | 348 | 271 | 77 |  |  |  |
| **Days with amphetamines use in past month (in treatment)** |  |  |  | 0.99 | 0.96, 1.01 | 0.3 |
| Mean, (SD) | 3.31, (7.57) | 3.53, (7.77) | 2.53, (6.84) |  |  |  |
| Median, [IQR] | 0.00, [0.00, 2.00] | 0.00, [0.00, 2.00] | 0.00, [0.00, 0.00] |  |  |  |
| Missing | 2 | 1 | 1 |  |  |  |
| **Fagerström Test for Nicotine Dependence (FTND) Score** |  |  |  | 1.17 | 1.06, 1.30 | **0.003** |
| Mean, (SD) | 3.68, (2.29) | 3.39, (2.24) | 4.57, (2.24) |  |  |  |
| Median, [IQR] | 4.00, [2.00, 5.00] | 3.00, [2.00, 5.00] | 5.00, [3.00, 6.00] |  |  |  |
| Missing | 72 | 68 | 4 |  |  |  |
| **Fagerström Test for Nicotine Dependence (including non-smokers as zeroes)** |  |  |  | 1.23 | 1.12, 1.35 | **<0.001** |
| Mean, (SD) | 3.23, (2.47) | 2.88, (2.39) | 4.45, (2.32) |  |  |  |
| Median, [IQR] | 3.00, [1.00, 5.00] | 3.00, [0.00, 5.00] | 5.00, [3.00, 6.00] |  |  |  |
| Missing | 6 | 5 | 1 |  |  |  |
| **Estimated $ spent on drugs in past 30 days** |  |  |  | 1.00 | 1.00, 1.00 | 0.091 |
| Mean, (SD) | 2,866.42, (4,643.51) | 2,644.92, (4,183.35) | 3,634.43, (5,927.89) |  |  |  |
| Median, [IQR] | 2,000.00, [1,000.00, 3,000.00] | 2,000.00, [1,000.00, 3,000.00] | 2,050.00, [1,000.00, 4,000.00] |  |  |  |
| **Number of days in the past 30 where Drug Problems were experienced** |  |  |  | 1.02 | 0.99, 1.04 | 0.2 |
| Mean, (SD) | 11.47, (13.57) | 10.79, (13.47) | 13.83, (13.70) |  |  |  |
| Median, [IQR] | 2.00, [0.00, 30.00] | 1.00, [0.00, 30.00] | 8.50, [0.00, 30.00] |  |  |  |
| **How troubled patient was by any drug problems experienced in past 30 days** |  |  |  |  |  |  |
| Not at all | 257 (47%) | 218 (52%) | 39 (32%) | — | — |  |
| Slightly | 51 (9.4%) | 26 (6.1%) | 25 (20%) | 5.26 | 1.90, 14.6 | **0.001** |
| Moderately | 73 (13%) | 57 (13%) | 16 (13%) | 1.55 | 0.62, 3.84 | 0.3 |
| Considerably | 84 (15%) | 65 (15%) | 19 (16%) | 1.56 | 0.66, 3.67 | 0.3 |
| Extremely | 80 (15%) | 57 (13%) | 23 (19%) | 2.32 | 0.96, 5.59 | 0.061 |
| **Whether patients were troubled to any extent by any drug problems experienced in past 30 days** |  |  |  |  |  |  |
| No | 257 (47%) | 218 (52%) | 39 (32%) | — | — |  |
| Yes | 288 (53%) | 205 (48%) | 83 (68%) | 2.21 | 1.21, 4.01 | **0.009** |
| **MAP Psychological Health Score** |  |  |  | 1.04 | 1.01, 1.08 | **0.007** |
| Mean, (SD) | 8.49, (7.37) | 7.68, (6.95) | 11.29, (8.12) |  |  |  |
| Median, [IQR] | 6.00, [2.00, 13.00] | 5.00, [2.00, 12.00] | 10.50, [5.00, 16.00] |  |  |  |
| **MAP Physical Health Score** |  |  |  | 1.04 | 1.01, 1.07 | **0.007** |
| Mean, (SD) | 13.57, (7.50) | 12.87, (7.55) | 15.95, (6.85) |  |  |  |
| Median, [IQR] | 13.00, [8.00, 19.00] | 12.00, [7.00, 19.00] | 16.00, [12.00, 20.00] |  |  |  |
| Missing | 51 | 41 | 10 |  |  |  |
| **Average Dose Prescribed per session in past 30 days** |  |  |  | 1.00 | 0.99, 1.00 | 0.082 |
| Mean, (SD) | 172.86, (84.72) | 175.38, (85.30) | 164.08, (82.43) |  |  |  |
| Median, [IQR] | 155.00, [110.00, 225.00] | 160.00, [112.56, 233.33] | 143.61, [104.17, 203.87] |  |  |  |
| Missing | 7 | 5 | 2 |  |  |  |
| **Average Daily Dose Prescribed in past 30 days** |  |  |  | 1.00 | 1.00, 1.00 | 0.6 |
| Mean, (SD) | 562.59, (261.58) | 555.93, (257.78) | 585.91, (274.28) |  |  |  |
| Median, [IQR] | 540.00, [360.00, 723.30] | 540.00, [360.00, 720.00] | 540.00, [375.00, 760.00] |  |  |  |
| Missing | 5 | 3 | 2 |  |  |  |
| **Average Dose Used per session in past 30 days** |  |  |  | 1.00 | 0.99, 1.00 | 0.13 |
| Mean, (SD) | 170.42, (86.07) | 173.16, (86.25) | 160.82, (85.10) |  |  |  |
| Median, [IQR] | 150.00, [105.16, 225.00] | 156.19, [109.89, 231.01] | 137.09, [98.44, 205.39] |  |  |  |
| Missing | 10 | 7 | 3 |  |  |  |
| **Average Daily Dose Used in past 30 days** |  |  |  | 1.00 | 1.00, 1.00 | >0.9 |
| Mean, (SD) | 494.46, (246.10) | 492.88, (242.90) | 500.00, (258.03) |  |  |  |
| Median, [IQR] | 470.25, [287.58, 650.00] | 470.50, [287.36, 648.50] | 470.00, [290.17, 657.33] |  |  |  |
| Missing | 7 | 4 | 3 |  |  |  |
| **Communication Assessment Tool (CAT) Score** |  |  |  | 0.81 | 0.69, 0.95 | **0.009** |
| Mean, (SD) | 4.10, (1.09) | 4.20, (1.03) | 3.74, (1.23) |  |  |  |
| Median, [IQR] | 4.43, [3.71, 5.00] | 4.71, [3.93, 5.00] | 4.00, [2.98, 5.00] |  |  |  |
| Missing | 28 | 18 | 10 |  |  |  |
| **Patient's rating of doctor involving them in decisions as much as they wanted** |  |  |  | 0.80 | 0.70, 0.91 | **0.001** |
| Mean, (SD) | 3.99, (1.26) | 4.10, (1.19) | 3.60, (1.40) |  |  |  |
| Median, [IQR] | 4.00, [3.00, 5.00] | 5.00, [4.00, 5.00] | 4.00, [3.00, 5.00] |  |  |  |
| Missing | 16 | 11 | 5 |  |  |  |
| **Patient's rating of doctor involving them in decisions as much as they wanted (dichotomized)** |  |  |  |  |  |  |
| Less Than Excellent | 275 (52%) | 199 (48%) | 76 (65%) | — | — |  |
| Excellent | 254 (48%) | 213 (52%) | 41 (35%) | 0.65 | 0.47, 0.88 | **0.006** |
| Missing | 16 | 11 | 5 |  |  |  |
| **Visual Assessment Scale of 'Drug Liking'** |  |  |  | 0.98 | 0.97, 0.99 | **<0.001** |
| Mean, (SD) | 74.22, (24.17) | 78.08, (21.08) | 61.02, (29.05) |  |  |  |
| Median, [IQR] | 80.00, [60.00, 90.00] | 80.00, [70.00, 95.00] | 67.50, [50.00, 80.00] |  |  |  |
| Missing | 6 | 6 | 0 |  |  |  |
| ^1^ n (%) | | | | | | |
| ^2^ OR = Odds Ratio, CI = Confidence Interval | | | | | | |

Values are bolded if there is a significant (p<0.05) difference in means between dose-satisfaction groups (for continuous variables) or significant difference in distribution of dose-satisfaction status between one or more levels of a categorical variable and the (not bolded) reference level.

*p<0.05, **p<0.01, ***p<0.001

Abbreviations used: EQ5D-CAN = Canadian version of the EuroQol 5 Dimension descriptive system for health-related quality-of-life; OTI = Opiate Treatment Index; FTND = Fagerström Test of Nicotine Dependence; MAP = Maudsley Addiction Profile; VAS = Visual Assessment Scale

**Appendix Table 2.** Stepwise adjusted GEEGLM model of variables associated with patient-reported dose satisfaction status

|  | Patient-reported iOAT Dose Satisfaction | | | | | | | | | | | | | | | | | | |  |
| --- | --- | --- | --- | --- | --- | --- | --- | --- | --- | --- | --- | --- | --- | --- | --- | --- | --- | --- | --- | --- |
|  | Step 1 | | Step 2 | | Step 3 | | Step 4 | | | Step 5 | | | | | Step 6 | | | | | Step 7 |
|  | (socio-demographics) | | (Physical Health) | | (Mental Health) | | (Quality of Life) | | | (Substance Use and Treatment) | | | | | (Shared Decision Making) | | | | | (Drug Liking) |
|  | AOR (95%  CI) | AOR (95% CI) | | AOR (95% CI) | | | | AOR (95% CI) | | | AOR (95%  CI) | | | AOR (95% CI) | | | | AOR (95% CI) | | |
| **Socio-demographics** |  | |  | |  | |  | | |  | | | | |  | | | | |  |
| Age | 0.980 (0.946, 1.02) | 0.973 (0.939, 1.01) | | 0.983 (0.945, 1.02) | | | | 0.983 (0.942, 1.02) | | | 0.987 (0.949, 1.03) | | | 0.996 (0.955, 1.04) | | | | 0.996 (0.960, 1.03) | | |
| *Gender* |  | |  | |  | |  | | |  | | | | |  | | | | |  |
| Woman |  | |  | |  | |  | | |  | | | | |  | | | | |  |
| Man | 0.725 (0.355, 1.48) | 0.758 (0.371, 1.55) | | 0.809 (0.399, 1.64) | | | | 0.789 (0.384, 1.62) | | | 0.959 (0.463, 1.98) | | | 0.848 (0.409, 1.76) | | | | 0.818 (0.403, 1.66) | | |
| *Indigeneity* |  | |  | |  | |  | | |  | | | | |  | | | | |  |
| Not Indigenous |  | |  | |  | |  | | |  | | | | |  | | | | |  |
| Indigenous (First Nations, Metis, and/or Inuit) | 1.32 (0.618, 2.80) | 1.27 (0.598, 2.71) | | 1.30 (0.607, 2.78) | | | | 1.34 (0.620, 2.89) | | | 1.42 (0.646, 3.14) | | | 1.50 (0.681, 3.29) | | | | 1.38 (0.669, 2.85) | | |
| *Education* |  | |  | |  | |  | | |  | | | | |  | | | | |  |
| Some High School or less |  | |  | |  | |  | | |  | | | | |  | | | | |  |
| High School Diploma | 1.17 (0.557, 2.46) | 1.29 (0.618, 2.71) | | 1.24 (0.587, 2.60) | | | | 1.32 (0.622, 2.79) | | | 1.43 (0.653, 3.13) | | | 1.33 (0.601, 2.96) | | | | 1.51 (0.711, 3.22) | | |
| At least some post-secondary | 0.749 (0.335, 1.67) | 0.838 (0.371, 1.89) | | 0.829 (0.377, 1.83) | | | | 0.879 (0.392, 1.97) | | | 1.01 (0.470, 2.18) | | | 0.911 (0.422, 1.97) | | | | 0.896 (0.430, 1.87) | | |
| **Physical Health** |  | |  | |  | |  | | |  | | | | |  | | | | |  |
| Opioid Treatment Index - Health Section Total Score (minus Gynecological subscale) |  | **1.01 (1.00, 1.02)** | | 1.01 (0.995, 1.02) | | | | 1.01 (0.995, 1.02) | | | 1.00 (0.992, 1.02) | | | 1.00 (0.990, 1.02) | | | | 1.01 (0.993, 1.02) | | |
| **Mental Health** |  | |  | |  | |  | | |  | | | | |  | | | | |  |
| At least one lifetime suicide attempt |  |  | | 1.65 (0.803, 3.40) | | | | 1.47 (0.706, 3.06) | | | 1.52 (0.756, 3.06) | | | 1.49 (0.726, 3.07) | | | | 1.39 (0.686, 2.81) | | |
| MAP Psychological Score |  |  | | 1.03 (0.989, 1.08) | | | | 1.03 (0.987, 1.07) | | | 1.03 (0.983, 1.07) | | | 1.02 (0.980, 1.07) | | | | 1.01 (0.976, 1.04) | | |
| **Quality of Life** |  | |  | |  | |  | | |  | | | | |  | | | | |  |
| EQ5D-CAN Score |  | |  | | |  | | | 0.769 (0.165, 3.58) | | | 0.679 (0.150, 3.08) | | | | 0.664 (0.142, 3.10) | | | 0.998 (0.224, 4.44) | |
| **Substance Use and Treatment** |  | |  | |  | |  | | |  | | | | |  | | | | |  |
| Average Daily Prescribed iOAT dose* |  | |  | | |  | | |  | | | 1.00 (0.999, 1.00) | | | | 1.00 (0.999, 1.00) | | | 1 (0.999, 1.00) | |
| Number of days (out of past 30 days) that client used criminalized opioids ("heroin") (in treatment) |  | |  | |  | |  | | | 1.02 (0.994, 1.04) | | | 1.02 (0.996, 1.05) | | | | 1.00 (0.974, 1.04) | | | |
| Number of days (out of past 30 days) that client smoked crack cocaine |  | |  | |  | |  | | | 1.02 (0.990, 1.05) | | | 1.01 (0.985, 1.04) | | | | 1.01 (0.986, 1.04) | | | |
| Current Smoker (versus Non-Smoker) |  | |  | |  | |  | | | **3.69 (1.23, 11.1)** | | | **3.24 (1.15, 9.14)** | | | | **1.17 (1.06, 1.30)** | | | |
| Client reports feeling troubled (versus untroubled) with problems associated with criminalized drug use |  | |  | |  | |  | | | **1.92 (1.05, 3.50)** | | | **2.04 (1.10, 3.76)** | | | | **2.06 (1.12, 3.79)** | | | |
| **Shared Decision Making** |  | |  | |  | |  | | |  | | | | |  | | | | |  |
| *Client's perspective of doctor involving them in decisions as much as the client wants (dichotomized)* |  | |  | |  | |  | | |  | | | | |  | | | | |  |
| "Less than excellent" |  | |  | |  | |  | | |  | | | | |  | | | | |  |
| "Excellent" |  | |  | |  | |  | | |  | | | | | **0.652 (0.453, 0.938)** | | | | | 0.814 (0.614, 1.08) |
| **Drug Liking** |  | |  | |  | |  | | |  | | | | |  | | | | |  |
| Visual Assessment Scale of "Liking the (prescribed) Drug" |  | |  | |  | |  | | |  | | | | |  | | | | | **0.983 (0.973, 0.993)** |
| **QIC** | 584.4 | | 568 | | 558.6 | | 550.6 | | | 532.9 | | | | | 515 | | | | | 477.3 |
| *Adjusted Odds Ratios (AOR) show the odds of a change in the dependent variable (i.e. from "0", satisfied with dose, to "1", wants a higher dose) per 1 unit change in the independent variable (continuous variable) or for one level of a categorical variable compared to the reference group (the first level of the categorical variable), holding all other variables constant.* | | | | | | | | | | | | | | | | | | | | |

Values are bolded if there is a significant (p<0.05) difference in means between dose-satisfaction groups (for continuous variables) or significant difference in distribution of dose-satisfaction status between one or more levels of a categorical variable and the (not bolded) reference level. *p<0.05, **p<0.01, ***p<0.001

Abbreviations used: EQ5D-CAN = Canadian version of the EuroQol 5 Dimension descriptive system for health-related quality-of-life; OTI = Opiate Treatment Index; FTND = Fagerström Test of Nicotine Dependence; MAP = Maudsley Addiction Profile; VAS = Visual Assessment Scale
